# Abnormality of Hair Growth with Total Absence of Teeth

**Published:** 1899-05

**Authors:** 


### Abnormality of Hair-Growth With Total Absence of Teeth.

That there is some relationship between the growth of the hair and the teeth has long been known. Dr. Guilford now reports the case of a man forty-eight years old, who was, and had always been, toothless. He also entirely lacked the sense of smell, and almost that of taste. There was no deformity in the bones of the jaws. His beard was abundant, as was also the hair in axillae and on pubis. His head was covered with a spare but soft and downy growth, similar to that seen in early childhood. His body otherwise was entirely devoid of hair, even of laungo hairs. As he never perspired it would seem that there was some deficiency in the sweating apparatus. His maternal grandmother and uncle never had either teeth or hair. The patient was one of twenty-one children, and the only one who was toothless, though some of his brothers lacked certain teeth. —*Mass. Med. Jour.*, 1893, *No.* 13, *page* 484.

